# Determinants of Midwifery Workforce Disaster Preparedness and Its Impact on the Continuity of Maternal Care: A Systematic Review

**DOI:** 10.3390/healthcare14111499

**Published:** 2026-05-28

**Authors:** Eirini Orovou, Alina Liepinaitienė, Chrysoula Taskou, Kleanthi Gourounti, Dimitrios Papoutsis, Antigoni Sarantaki

**Affiliations:** 1 Midwifery Department, University of Western Macedonia, 50200 Kozani, Greece; 2 Department of Environmental Sciences, Faculty of Natural Sciences, Vytautas Magnus University, 44248 Kaunas, Lithuania; alina.liepinaitiene@vdu.lt; 3 School of Heal Sciences, SMK College of Applied Sciences, 46326 Kaunas, Lithuania; 4 Midwifery Department, International Hellenic University, 57400 Thessaloniki, Greece; chrysa_t85@hotmail.com; 5 Midwifery Department, Faculty of Health and Care Sciences, University of West Attica, 12243 Athens, Greece; 6 Faculty of Medicine, Kauno Kolegija Higher Education Institution, 50468 Kaunas, Lithuania

**Keywords:** midwifery workforce, disaster preparedness, integrated care, health equity, climate resilience, maternal-child health

## Abstract

**Highlights:**

**What are the main findings?**

- Midwifery workforce preparedness is a multidimensional construct influenced by individual competencies, organizational readiness, and psychosocial resilience, directly affecting response capacity during disasters.
- Insufficient disaster-specific training, role ambiguity, and limited institutional preparedness are consistently linked to disruptions in antenatal, intrapartum, and postnatal care continuity.

**What are the implications of the main findings?**

- Integrating disaster education, simulation-based training, and clear role delineation into midwifery practice is essential to strengthen workforce preparedness and service continuity.
- Health systems should formally incorporate midwives into disaster planning and enhance organizational support mechanisms to improve maternal care resilience during climate-related emergencies.

**Abstract:**

Background/Objectives: Natural disasters and climate-related emergencies increasingly disrupt maternal healthcare systems, placing growing demands on the midwifery workforce. While midwives play a critical role in maintaining continuity of care, evidence on how workforce preparedness influences service delivery remains limited. This systematic review aimed to synthesize evidence on determinants of midwives’ disaster preparedness and examine their association with continuity of maternal care. Methods: A systematic review was conducted in accordance with PRISMA 2020 guidelines. Searches were performed in PubMed, Embase, CINAHL, Scopus, and Web of Science from inception to March 2026. Eligible studies examined midwives or midwifery-led care in natural disasters or climate-related emergencies. Data were extracted independently by two reviewers, and methodological quality appraised using the Mixed Methods Appraisal Tool (MMAT). Due to substantial methodological and clinical heterogeneity across study designs, populations, and outcomes, a meta-analysis was not feasible and findings were synthesized narratively. Results: Nine studies met the inclusion criteria, with the evidence base consisting predominantly of qualitative and cross-sectional studies, alongside one cohort study. Evidence was mainly derived from earthquake-affected settings. Preparedness was influenced by individual, professional, organizational, and psychosocial factors. Insufficient disaster-specific training, role ambiguity, and limited institutional preparedness were linked to reduced response capacity and disruptions across antenatal, intrapartum, and postnatal care. Conclusions: The evidence suggests that midwifery workforce preparedness is an important determinant of continuity of maternal care during disasters and climate-related emergencies. Strengthening disaster education, integrating midwives into emergency planning, and enhancing organizational support are essential to improve health system resilience. Further longitudinal and intervention-based research across diverse disaster contexts is needed to strengthen the evidence base. However, the findings should be interpreted cautiously due to the limited number and heterogeneity of included studies.

## 1. Introduction

Natural disasters and climate-related extreme events are increasingly disrupting health systems worldwide, exposing structural vulnerabilities in essential service delivery [1,2]. Among the services most sensitive to systemic disruption is maternal care [3]. Antenatal monitoring, skilled birth attendance, emergency obstetric interventions, and postnatal follow-up depend on continuity, coordination, and a stable skilled workforce [4,5]. However, when disasters compromise infrastructure, transportation, supply chains, and staffing capacity, the sustainability of maternal services becomes precarious [6].

Addressing maternal health during disasters is intrinsically linked to health equity, as environmental crises tend to disproportionately affect marginalized communities with limited resource access. Midwives play a crucial role as the primary facilitators of integrated care, effectively bridging the gap between community-based support and acute obstetric interventions, and they form a fundamental pillar of maternal and newborn care on a global scale. Across community and facility-based settings, they ensure continuity of care throughout pregnancy, childbirth, and the postpartum period [7]. In disaster contexts, midwives frequently operate under conditions of infrastructure damage, workforce shortages, resource scarcity, and heightened psychosocial strain. Their capacity to sustain maternal services during such crises is not solely dependent on clinical competence but also on preparedness, professional resilience, institutional support, and systemic integration into disaster planning frameworks [8,9]. In the context of this review, integrated care refers to the coordinated continuity of maternal services across antenatal, intrapartum, and postnatal care settings during disasters and climate-related emergencies. Health equity relates to the ability of disaster-response systems to maintain accessible and consistent maternity care for vulnerable and resource-limited populations. Midwifery workforce preparedness is conceptualized as a key enabling factor supporting both integrated care continuity and equitable maternal health access under crisis conditions. Within this framework, preparedness refers to the competencies, training, and organizational readiness required before and during disaster response, resilience reflects the capacity of the workforce to sustain functioning under prolonged crisis conditions, and continuity of care represents the ability of maternity services to remain accessible and coordinated throughout disaster exposure.

Preparedness in midwifery practice extends beyond technical knowledge of emergency protocols [10]. It encompasses disaster-specific training, simulation exposure, clarity of role within emergency response structures, access to protective resources, psychological readiness, and confidence in organizational leadership [11]. Furthermore, socio-contextual determinants (including caregiving responsibilities, concerns about family safety, and perceived institutional protection) may influence willingness to respond and sustained engagement during prolonged emergencies [12]. Previous reviews on disaster preparedness have primarily focused on general nursing workforce readiness, emergency response systems, or maternal and neonatal outcomes during disasters. However, limited attention has been given specifically to the midwifery workforce and its role in sustaining continuity of maternal care during environmental crises. Moreover, the relationship between midwifery preparedness, integrated care continuity, and equitable maternal healthcare access has not been systematically synthesized. The present review seeks to address this gap by integrating workforce preparedness and maternal care continuity perspectives within disaster and climate-related emergency settings.

Despite recognition of maternal health vulnerability in disaster settings, the preparedness and resilience of the midwifery workforce remain insufficiently synthesized in the literature. Existing studies often evaluate self-reported readiness without systematically examining how preparedness determinants interact with real-world continuity of maternal care [8,13]. Moreover, workforce sustainability (particularly in the face of repeated climate-related events) has not been adequately integrated into disaster preparedness discussions [14].

Strengthening midwifery resilience is not merely a matter of emergency planning; it is a strategic investment in safeguarding maternal and neonatal outcomes [15]. Understanding the factors that enable midwives to maintain continuity of care under crisis conditions is essential for health system resilience, workforce retention, and equitable maternal health protection in disaster-prone regions [16].

The review question guiding: *How do determinants of midwifery workforce disaster preparedness influence the continuity of maternal care during natural disasters and climate-related emergencies?* Accordingly, this review aimed to synthesize evidence on: (a) the determinants of midwives’ disaster preparedness and response capacity, and (b) the impact of natural disasters and climate-related emergencies on continuity of maternal care. By integrating workforce and service-continuity perspectives, the review sought to identify factors that enable or constrain midwives’ ability to sustain maternity care during environmental crises.

## 2. Materials and Methods

### 2.1. Study Design and Reporting Standards

This systematic review was conducted in accordance with the Preferred Reporting Items for Systematic Reviews and Meta-Analyses (PRISMA 2020) guidelines [17]. The review protocol was prospectively registered in PROSPERO (CRD420261352549).

### 2.2. Eligibility Criteria

Studies were considered eligible if they focused on midwives or midwifery-led care in disaster contexts. Relevant scenarios included earthquakes, floods, hurricanes, cyclones, storms, wildfires, heatwaves, and other natural emergencies. Publications involving mixed healthcare samples were included if they allowed for the extraction of midwife-specific findings or addressed maternity-service continuity relevant to midwifery. The inclusion of such studies was limited to those with disaggregated midwifery data or findings that affect maternity-service continuity. Studies involving pregnant women were considered only if the intervention represented a midwifery-led continuity-of-care model pertinent to the review. Similarly, studies involving pregnant women were included only if they evaluated midwifery-led models, providing essential evidence on workforce structures mitigating disaster-related stress. These studies were included because continuity-of-care models provided indirect but relevant evidence regarding organizational preparedness, workforce resilience, and the capacity of midwifery-led systems to sustain maternal care during disaster conditions.

Editorials, commentaries, conference abstracts lacking comprehensive data, and single case reports were excluded. Studies published in English up to 29 March 2026 were considered. Eligibility criteria were established a priori according to objectives related to population, exposure/context, outcomes, and study design.

Two interrelated outcome domains were examined: (a) determinants of midwives’ disaster preparedness and workforce resilience, including readiness, competence, disaster-related training exposure, willingness to respond, perceived organizational preparedness, role clarity, leadership integration, and psychological factors influencing response capacity, (b) continuity of maternal care during disasters, encompassing disruptions to antenatal, intrapartum, and postnatal services, workforce shortages affecting maternity care, service closures or relocation of maternity units, barriers to emergency obstetric referral, and impacts on midwifery-led models of care. Maternal or neonatal health outcomes were included when directly linked to service disruption.

Quantitative (cross-sectional, cohort, quasi-experimental, interrupted time-series) studies, qualitative studies, and mixed-methods studies were considered eligible. No date restrictions were applied during the search. Studies published in English up to 29 March 2026 were considered eligible for inclusion.

### 2.3. Search Strategy

A systematic and comprehensive literature search was conducted across five major electronic databases: MEDLINE (via PubMed), Embase, CINAHL, Scopus, and Web of Science. The search spanned each database’s inception through 29 March 2026. Furthermore, we manually examined the reference lists of the included studies to identify additional relevant publications. To mitigate publication bias, grey literature was searched using the official repositories of the World Health Organization (WHO) and the International Confederation of Midwives (ICM). Search terms included ‘midwifery disaster guidelines’, ‘maternal health emergency protocols’, and ‘workforce resilience reports’ to identify non-peer-reviewed strategic mandates and training frameworks. Grey literature searches focused on policy documents, strategic frameworks, workforce preparedness reports, and maternal health emergency guidance relevant to midwifery practice during disasters. Searches were conducted manually within the official WHO and ICM repositories using combinations of terms including ‘midwifery disaster preparedness’, ‘maternal health emergency care’, ‘continuity of care’, ‘climate resilience’, and ‘workforce preparedness’. Grey literature sources were screened according to the same conceptual relevance criteria applied to peer-reviewed studies.

The search strategy was structured around three thematic pillars: (1) the midwifery and nursing workforce, (2) natural disasters and climate-induced emergencies, and (3) clinical preparedness, professional resilience, and the continuity of integrated maternal/perinatal care. To ensure high sensitivity, the strategy employed a combination of Medical Subject Headings (MeSH), Emtree terms, and CINAHL headings, alongside a wide array of free-text keywords and truncated terms, utilizing Boolean operators ‘AND’ and ‘OR’. For the disaster component, specific hazards such as ‘earthquake*’, ‘flood*’, and ‘wildfire*’ were included to capture diverse geographical contexts. Regarding maternity care, the search focused on ‘integrated care’ and ‘equity in access’ through terms like ‘continuity of care’ and ‘perinatal care’.

The full PubMed search strategy is provided below as an example and was adapted for each database using appropriate indexing terms: (midwife*[Title/Abstract] OR “nurse-midwife*”[Title/Abstract] OR “certified professional midwife*”[Title/Abstract] OR “maternity nurse*”[Title/Abstract]) AND (disaster*[Title/Abstract] OR “natural disaster*”[Title/Abstract] OR earthquake*[Title/Abstract] OR flood*[Title/Abstract] OR hurricane*[Title/Abstract] OR cyclone*[Title/Abstract] OR storm*[Title/Abstract] OR wildfire*[Title/Abstract] OR heatwave*[Title/Abstract] OR “extreme weather”[Title/Abstract] OR “climate-related emergency*”[Title/Abstract]) AND (preparedness [Title/Abstract] OR readiness [Title/Abstract] OR competence [Title/Abstract] OR training [Title/Abstract] OR resilience [Title/Abstract] OR continuity of care [Title/Abstract] OR maternal care [Title/Abstract] OR maternity care [Title/Abstract] OR “perinatal care”[Title/Abstract] OR “obstetric care”[Title/Abstract]).

### 2.4. Study Selection

All records retrieved from MEDLINE (via PubMed), Embase, CINAHL, Scopus, and Web of Science were exported into reference management software, and duplicates were removed prior to screening. Two reviewers independently screened titles and abstracts against the predefined eligibility criteria. Full texts of potentially relevant articles were then retrieved and assessed independently. Disagreements at any stage were resolved through discussion. The study selection process is presented in the PRISMA 2020 flow diagram [17].

### 2.5. Data Extraction

Data were extracted independently by two reviewers using a standardized form. Extracted variables included study characteristics (author, year, country, design, setting), population details, disaster type, preparedness-related determinants, continuity-of-care outcomes, and key findings. Qualitative themes and quantitative findings were extracted separately and then integrated during narrative synthesis to preserve methodological integrity while enabling cross-study comparison. Discrepancies were resolved through discussion and consensus.

### 2.6. Risk of Bias

Risk of bias was assessed using the Mixed Methods Appraisal Tool (MMAT, 2018) [18]. Two reviewers independently appraised each study according to the design-specific criteria. Methodological limitations were reported descriptively by study domain and design. To align with MMAT guidance, no single aggregated quality score was used to determine study inclusion or exclusion.

### 2.7. Approach to Interpreting Confidence in the Evidence

Given the predominance of qualitative and cross-sectional evidence, together with substantial heterogeneity in study designs, disaster contexts, outcome measures, and reporting approaches, the application of a formal certainty-of-evidence framework such as GRADE was considered inappropriate for this review. Instead, confidence in the body of evidence was interpreted narratively by considering methodological limitations, consistency of findings across studies, contextual relevance, and transferability of findings across disaster settings.

### 2.8. Data Synthesis

Due to the anticipated heterogeneity in study designs, measurement tools, and outcome definitions, a primarily narrative synthesis approach was undertaken. Findings were systematically organized into two overarching analytical streams: (a) determinants of midwives’ disaster preparedness and workforce resilience, and (b) patterns of disruption affecting continuity of maternal care during natural disasters and climate-related emergencies. Preparedness-related findings were synthesized across individual, professional, organizational, and socio-contextual domains to identify recurring determinants influencing response capacity and workforce sustainability. Disruptions in maternal care were categorized according to phases of the care continuum (antenatal, intrapartum, and postnatal) to clarify where and how service continuity was compromised. Due to substantial methodological and clinical heterogeneity among the included studies, meta-analysis was not feasible and findings were synthesized narratively.

## 3. Results

### 3.1. Study Selection

**Figure 1:**
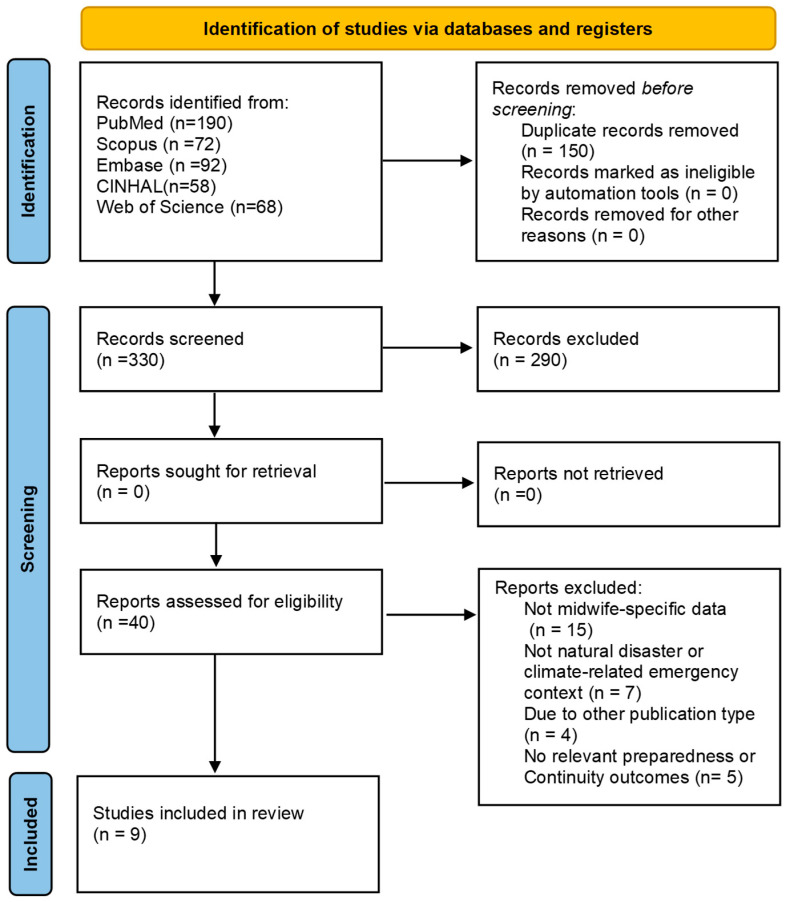
PRISMA 2020 flow diagram of the study selection process.

The database search identified a total of 480 records. After removal of duplicates, 330 records were screened by title and abstract. Of these, 40 full-text articles were assessed for eligibility, and 9 studies met the inclusion criteria. The study selection process is presented in Figure 1.

### 3.2. Study Characteristics

Nine studies met the inclusion criteria, including six qualitative studies, two cross-sectional studies, and one prospective cohort study, encompassing diverse geographical contexts including Iran, Turkey, Australia, the United States, Indonesia, and South Africa. Study designs included five qualitative studies, three cross-sectional surveys, and one prospective cohort study. Sample sizes ranged from small qualitative samples (*n* = 12–19) to larger survey-based samples exceeding 300 participants. Disaster contexts included earthquakes, floods, hurricanes, severe storms, and other natural disasters. Earthquake-related contexts were most frequently represented, particularly in studies conducted in Iran and Turkey. The majority of the studies included in the analysis primarily focused on examining the determinants of midwifery workforce preparedness, encompassing disaster-related competencies, organizational readiness, and psychosocial resilience. In contrast, a smaller number of studies evaluated continuity-of-care outcomes, such as maintenance of antenatal, intrapartum, and postnatal services during disaster exposure. Detailed characteristics are presented in Table 1.

### 3.3. Methodological Quality

The prevalence of qualitative and cross-sectional research designs poses challenges for establishing causal relationships and limits the applicability of findings across different disaster scenarios. The methodological quality was appraised using the MMAT [18]. Overall, the studies reviewed presented low to moderate methodological concerns. Most qualitative studies showed strong coherence between research questions, data collection procedures, and interpretation of findings. Studies described as having minor or moderate methodological concerns generally demonstrated limitations related to sampling strategies, representativeness, reflexivity, completeness of outcome data, or clarity of analytic procedures, depending on study design and MMAT domain. Minor concerns were identified in some studies regarding reflexivity and clarity of analytic procedures. Among quantitative studies, moderate methodological concerns primarily related to sampling representativeness and potential nonresponse bias. The cohort study demonstrated comparatively higher methodological rigor, including appropriate outcome measurement and follow-up procedures. A summary of the methodological appraisal is presented in Table 2. Detailed MMAT criterion-level assessments are presented in Supplementary Table S1.

### 3.4. Determinants of Midwives’ Disaster Preparedness and Response Capacity

Τhe following findings are presented according to the two principal analytical domains of the review: workforce preparedness determinants and continuity-of-care outcomes. Across the included studies, disaster preparedness was shaped by interacting determinants at the individual, professional, organizational, and psychosocial levels. Several studies identified gaps in disaster-specific knowledge and clinical competencies, particularly in neonatal emergency management and referral stabilization. Cross-sectional evidence from Iran reported moderate self-perceived competency levels, with nearly half of the participants reporting only average preparedness [19]. Disaster exposure experience and higher educational attainment were associated with higher self-reported perceived competence [22]. Qualitative findings further emphasized the need for structured disaster training and simulation-based learning to enhance skill confidence and response readiness [8].

Findings from qualitative studies suggested that organizational preparedness may represent an important determinant of workforce capacity [23]. Studies conducted in Turkey and South Africa highlighted insufficient institutional planning, unclear role delineation, and limited access to structured disaster protocols [23,24]. Midwives reported limited integration into formal disaster response frameworks and reduced visibility in emergency planning structures [25]. Qualitative findings further suggested that limited professional visibility within disaster-response systems may influence role clarity, participation in decision-making processes, and access to organizational support during crises. These factors appeared to affect both workforce resilience and continuity of maternity care delivery in resource-constrained disaster settings. Qualitative findings suggested that psychological strain, emotional burden, and concerns regarding personal and family safety may influence response sustainability. Earthquake-related qualitative studies described emotional exhaustion, moral distress, and heightened stress under prolonged crisis conditions [8,21,23]. Perceived organizational support and peer collaboration were identified as protective factors enhancing resilience.

Research indicates that the exclusion of midwives from formal emergency response frameworks and their low occupational status significantly hinder effective midwifery practice [25]. Professional equity is undermined when midwives, despite their crucial frontline roles, are not visible in leadership positions within emergency planning.

### 3.5. Continuity of Maternal Care During Natural Disasters

The determinants of preparedness identified in the previous section were closely reflected in patterns of service disruption during disasters. Insufficient training, limited organizational preparedness, and workforce strain were frequently linked to challenges in maintaining continuity of maternal care across antenatal, intrapartum, and postnatal services. Qualitative accounts from earthquake-affected regions described shortages of essential supplies, damaged infrastructure, and workforce redistribution [25]. Referral systems were compromised, particularly for neonatal complications requiring stabilization and transfer [23]. Several qualitative studies reported that disaster conditions frequently required midwives to assume expanded clinical and coordination roles within resource-constrained environments. Qualitative evidence from earthquake-affected settings described increased workload, shortages of essential supplies, and organizational gaps that limited effective service delivery [23,25]. Participants reported that insufficient disaster preparedness planning and limited surge capacity placed additional strain on the maternity workforce and contributed to psychological and emotional burden. In contrast, evidence from Australia indicated that structured midwifery continuity-of-care models may offer protective effects during disaster exposure. In a cohort study following severe flooding, women receiving continuity-of-care midwifery services showed reduced adverse infant neurodevelopment outcomes despite prenatal disaster stress [20].

## 4. Discussion

To our knowledge, this review is among the first to systematically examine the integration of midwifery workforce preparedness and continuity of maternal care in disaster settings. It synthesizes evidence on the determinants of midwives’ preparedness and explores how these factors influence the maintenance of maternal care during natural disasters and climate-related emergencies. Across the included studies, preparedness of the midwifery workforce emerged as a multidimensional construct influenced by individual competencies, organizational preparedness, and psychosocial resilience [19,22,24]. While midwives demonstrated strong professional commitment to maintaining maternal services during crises [21], recurring gaps were identified in disaster-specific training, institutional preparedness, and workforce support mechanisms. These gaps were frequently reflected in disruptions to antenatal, intrapartum, and neonatal care delivery during disaster conditions [20,25].

The available evidence suggests a possible association between workforce preparedness determinants and the stability of maternal services. Specifically, individual gaps in disaster-specific training and neonatal emergency management appeared to be associated with disruptions in postnatal care and referral stabilization. At the organizational level, role ambiguity and limited inclusion of midwives within formal disaster planning frameworks appeared to function as barriers that compromised intrapartum care delivery and increased psychological strain. Conversely, structured midwifery-led continuity models—a form of organizational preparedness—appeared to demonstrate potentially protective effects, maintaining maternal-infant health outcomes even during severe environmental stressors. These findings suggest that preparedness may function as an upstream determinant of workforce resilience, which in turn may influence continuity of maternal care during disasters.

Findings from the included studies suggest that disaster preparedness among midwives extends beyond technical competence and requires integration of training, role clarity, and organizational support [8,24]. Several studies reported moderate levels of self-perceived preparedness and significant gaps in disaster-specific competencies, particularly in neonatal emergency management and referral stabilization [19,22]. These findings align with broader literature emphasizing the need for structured disaster education and simulation-based training for maternity care providers [1,2,26].

Included qualitative studies suggested that disasters may disrupt multiple components of the maternal care continuum, including antenatal monitoring, referral systems, and facility-based delivery services [23,25]. Qualitative evidence from earthquake-affected regions suggested that shortages of essential supplies, infrastructure damage, and workforce redistribution often compromise the ability of midwives to maintain routine maternity services [23,25]. However, evidence from continuity-of-care models suggests that sustained relationships between midwives and women may mitigate some adverse effects of disaster-related stress and service disruption [27].

### 4.1. Implications for Policy and Training

The following implications are based both on findings derived from the included studies and on broader interpretive considerations informed by the wider literature on disaster preparedness and maternal health resilience. To ensure continuity, health systems should consider formally incorporating midwifery-led models into disaster-response frameworks, guaranteeing that maternal care remains seamless between community and facility settings. Strengthening workforce resilience may require the active involvement of midwives in the creation of disaster protocols, rather than treating them as passive responders. Addressing role ambiguity and offering structured psychosocial may contribute to improving professional equity, enabling midwives to continue caring for the most vulnerable women during climate-related events.

The findings suggest that strengthening midwifery preparedness requires multi-level intervention. The findings suggest that disaster competencies could be integrated into pre-service curricula and continuing professional development; simulation-based and interprofessional training may be beneficial components of preparedness programs; and greater inclusion of midwives within institutional and national disaster-response planning may strengthen preparedness capacity into institutional and national disaster-response planning. This approach is also supported by international guidance from the WHO, the United Nations Population Fund (UNFPA) [28], and the International Confederation of Midwives (ICM) [29], which emphasize the importance of disaster-ready and resilient midwifery systems for safeguarding continuity of maternal and newborn care during humanitarian and climate-related emergencies [9,16,30,31].

In parallel, organizational measures such as role clarification, leadership visibility, psychosocial support, and surge-capacity planning may help support both workforce resilience and continuity of maternal care [8].

### 4.2. Strengths and Limitations

This review underscores several notable strengths. Initially, it adheres to established methodological standards for systematic reviews, including compliance with PRISMA guidelines and the implementation of a predefined protocol, thereby enhancing both transparency and reproducibility. The review employed a comprehensive and systematic search strategy across multiple major databases, utilizing both controlled vocabulary and free-text terms to maximize the sensitivity and scope of evidence retrieval. Furthermore, a novel and integrative perspective was introduced by explicitly linking midwifery workforce preparedness with the continuity of maternal care in the context of natural disasters and climate-related emergencies. This conceptual synthesis addresses a significant gap in the existing literature, where these areas are often examined in isolation.

Additionally, the inclusion of diverse study designs, such as qualitative, cross-sectional, and cohort studies, facilitated a comprehensive exploration of both experiential and outcome-based dimensions of preparedness and care continuity. This approach provided a deeper understanding of complex, context-dependent phenomena that are not easily captured through quantitative methods alone. Lastly, the review offers practical and policy-relevant insights by identifying key determinants of preparedness, highlighting systemic barriers, and outlining actionable implications for education, workforce planning, and disaster preparedness frameworks in maternal healthcare settings.

Nevertheless, this review also has several limitations that should be acknowledged. First, the inclusion of studies published exclusively in English may have resulted in language bias and the omission of relevant evidence from non-English-speaking contexts. Second, the possibility of publication bias cannot be excluded, as studies reporting null or negative findings may be underrepresented in the literature. Third, the evidence base was largely concentrated in specific disaster contexts, particularly earthquakes, which may limit the generalizability of the findings to other types of natural disasters and climate-related emergencies. The broad eligibility framework adopted in this review was necessary to capture the complexity of disaster preparedness and continuity of maternal care across diverse settings; however, it also contributed to conceptual heterogeneity among included studies. In addition, substantial contextual heterogeneity across healthcare systems, disaster types, geographical regions, and sociocultural environments may further limit direct comparability and transferability of findings. Moreover, the absence of standardized measures for assessing disaster preparedness and continuity-of-care outcomes across studies limited direct comparison between findings and reduced the feasibility of quantitative synthesis. Substantial methodological, clinical, and contextual heterogeneity across studies also precluded quantitative synthesis and meta-analysis.

A significant limitation is the nature of the current evidence base, which relies heavily on qualitative and cross-sectional designs. This methodological concentration prevents the establishment of a causal link between specific preparedness interventions and measurable maternal mortality or morbidity rates. A particularly important limitation is that most included studies did not directly evaluate the relationship between preparedness determinants and measurable maternal or neonatal outcomes. Consequently, several conclusions regarding continuity of maternal care are based on indirect associations and interpretive synthesis rather than direct outcome-based evidence. Furthermore, the evidence is disproportionately focused on earthquake contexts in specific regions like Iran and Turkey, which may limit the transferability of these findings to slow-onset climate crises, such as prolonged droughts or heatwaves, where the demands on the midwifery workforce differ significantly from acute traumatic events. In addition, the relatively limited geographical distribution of included studies may restrict the applicability of findings to regions with different healthcare infrastructures, disaster-response systems, and maternity-care models.

Finally, several included studies involved mixed populations of healthcare professionals, including nurses and midwives, with findings not always fully disaggregated for midwives specifically. Although only studies with extractable or directly relevant midwifery-related findings were included, this may have reduced the specificity of conclusions related exclusively to midwifery workforce preparedness.

## 5. Conclusions

This review suggests that midwifery workforce preparedness may be shaped by a complex interplay of individual clinical competencies, organizational readiness, and psychosocial resilience. Key determinants influencing response capacity include disaster-specific training, clarity of professional roles, and the level of institutional support provided to frontline staff. These factors may be associated with continuity of maternal care, particularly as natural disasters frequently disrupt infrastructure, staffing, and referral pathways. However, these findings must be interpreted with caution, as the evidence is derived from a limited set of nine heterogeneous studies, primarily qualitative and cross-sectional, with a predominant focus on earthquake disasters. While a definitive causal relationship cannot yet be established, the recurring themes of role ambiguity and insufficient training across these studies suggest a recurring association with service disruption. The findings suggest that strengthening disaster education and formally integrating midwives into disaster planning may represent potentially important strategies.

## Figures and Tables

**Table 1:** Characteristics of included studies.

Authors/ Year/Country	Study Design	Population	Exposure (Disaster Type)	Key Focus (Exposure Variable)	Outcomes (Preparedness/Continuity)	Main Findings
Taghizadeh et al., (2018) Iran [19]	Cross-sectional	361 midwives	Earthquakes, floods	Professional competency in neonatal care	Knowledge, skills, perceived importance; 49.8% moderate competency; gaps in neonatal emergency management	Moderate competency levels were reported, with critical gaps in neonatal emergency management; competency was positively linked to education and disaster experience
Simcock et al., (2018), Australia [20]	Cohort	Pregnant women	Floods	Midwifery continuity of care model	Infant neurodevelopment	Midwifery continuity mitigated adverse neurodevelopmental impact of prenatal disaster stress
Monteblanco et al., (2019) U.S. [21]	Qualitative	Out of hospital Midwives	Hurricanes, Floods, Earthquakes, Severe storms	Midwives’ preparedness and perceived barriers to disaster response	Preparedness, commitment to respond, perceived barriers	Strong commitment to respond; perceived preparedness through care model; barriers included limited social networks and low occupational status
Mirmohammad (2022), Iran [22]	Qualitative	19 midwives	Natural disasters (specific types not specified)	Professional competencies required for midwifery practice in disasters	Disaster-related reproductive health competencies	Six core competency domains identified: safe pregnancy, safe childbirth, women’s health care, contraception, violence/STI management, and infant care
Keleş (2023), Turkey [23]	Qualitative	15 midwives who volunteered during earthquakes	Two major earthquakes	Post-earthquake caregiving experiences	Barriers to care delivery, disaster preparedness gaps, psychological impact	Organizational gaps, limited disaster training, resource shortages, and psychological strain underscored the need for structured preparedness and resilience training
Pusporini et al., (2024) Indonesia [13]	Cross- sectional	66 pregnant women	Natural disasters, earthquakes, and floods	Role of midwives; adequacy of disaster management	Role of midwives; adequacy of disaster management	Adequate disaster management significantly supported a favorable midwife role
Horn, (2024), South Africa [24]	Qualitative	17 nurses and midwives	Natural disasters/disaster preparedness in obstetric unit	Disaster preparedness knowledge, attitudes, and needs among obstetric staff	Awareness, attitudes, readiness gaps, training needs	Participants were aware of disaster planning but lacked assertiveness and preparedness; frequent training, simulations, and inclusive planning were recommended to improve readiness
Özkan et al. (2025) Turkey [25]	Qualitative	12 midwives, 8 nurses	Earthquake	Perinatal care delivery and workforce experiences	Service disruption, system barriers, coping, preparedness needs	Perinatal services were disrupted by resource shortages and organizational challenges; participants highlighted emotional strain and the need for structured disaster preparedness
Şimşek Bulgulu et al. (2025), Turkey [8]	Qualitative	Practicing midwives	Earthquake context	Midwifery disaster preparedness and professional competence	Disaster preparedness, perceived competence, organizational support	Insufficient training and role ambiguity hindered preparedness; organizational support and psychological readiness enhanced response capacity

Notes: Study design includes qualitative, cross-sectional, and cohort studies. Population refers to study participants (midwives, pregnant women, or mixed samples including nurses and midwives where relevant data were extractable). Exposure indicates the type of natural disaster or climate-related emergency examined. Key focus refers to the primary preparedness-related construct or intervention assessed. Outcomes include measures related to midwives’ preparedness, response capacity, or continuity of maternal/perinatal care.

**Table 2:** Summary of Methodological Quality.

Authors/Year	Design	Overall Appraisal (Concerns)
Taghizadeh (2018) [19]	Cross-sectional	Moderate
Simcock (2018) [20]	Cohort	Low
Monteblanco (2019) [21]	Qualitative	Minor
Mirmohammad (2022) [22]	Qualitative	Low
Keleş (2023) [23]	Qualitative	Minor
Pusporini (2024) [13]	Cross-sectional	Moderate
Horn (2024) [24]	Qualitative	Minor
Özkan (2025) [25]	Qualitative	Low
Şimşek Bulgulu (2025) [8]	Qualitative	Low

## Data Availability

No new data were created or analyzed in this study. Data supporting the findings of this study are derived from publicly available published articles, which are cited within the manuscript.

## Supplementary Materials

The following supporting information can be downloaded at: https://www.mdpi.com/article/10.3390/healthcare14111499/s1, Table S1: MMAT (2018) Criterion-Level Appraisal of Included Studies.

## Abbreviations

The following abbreviation is used in this manuscript:

STI	sexually transmitted infections
