# Supplementary material for: Determinants of Midwifery Workforce Disaster Preparedness and Its Impact on the Continuity of Maternal Care: A Systematic Review
Source: Healthcare (Basel). 2026 May 28;14(11):1499. doi: 10.3390/healthcare14111499 (PMC13256163; doi:10.3390/healthcare14111499)
Supplement: Supplementary file 1 [file healthcare-14-01499-s001.zip › healthcare-4277457-supplementary.pdf]

Determinants of Midwifery Workforce Disaster Preparedness and Its Impact on the Continuity of Maternal Care: A Systematic Review

Supplementary Material

Supplementary Table S1. MMAT (2018) criterion-level appraisal of included studies.

**Study**	**Study Design**	**Screening Questions (S1–S2)**	**Criterion 1**	**Criterion 2**	**Criterion 3**	**Criterion 4**	**Criterion 5**
Taghizadeh et al. (2018)	Cross-sectional	Yes / Yes	Yes	Yes	No	Yes	No
Simcock et al. (2018)	Cohort	Yes / Yes	Yes	Yes	Yes	Yes	Yes
Monteblanco et al. (2019)	Qualitative	Yes / Yes	Yes	Yes	Yes	Yes	No
Mirmohammad (2022)	Qualitative	Yes / Yes	Yes	Yes	Yes	Yes	Yes
Keleş (2023)	Qualitative	Yes / Yes	Yes	Yes	No	Yes	No
Pusporini et al. (2024)	Cross-sectional	Yes / Yes	Yes	Yes	Yes	No	Yes
Horn (2024)	Qualitative	Yes / Yes	Yes	Yes	Yes	Yes	No
Özkan et al. (2025)	Qualitative	Yes / Yes	Yes	Yes	Yes	Yes	Yes
Şimşek Bulgulu et al. (2025)	Qualitative	Yes / Yes	Yes	Yes	Yes	Yes	Yes
